# Loss of Growth Differentiation Factor 11 Shortens Telomere Length by Downregulating Telomerase Activity

**DOI:** 10.3389/fphys.2021.726345

**Published:** 2021-09-13

**Authors:** Di-Xian Wang, Xu-Dong Zhu, Xiao-Ru Ma, Li-Bin Wang, Zhao-Jun Dong, Rong-Rong Lin, Yi-Na Cao, Jing-Wei Zhao

**Affiliations:** ^1^Department of Pathology and Department of Human Anatomy, Histology, and Embryology, Sir Run Run Shaw Hospital, NHC and CAMS Key Laboratory of Medical Neurobiology, Zhejiang University School of Medicine, Hangzhou, China; ^2^Institute of Ageing Research, Hangzhou Normal University School of Medicine, Hangzhou, China; ^3^The General Hospital of Ningxia Medical University, Yinchuan, China

**Keywords:** growth differentiation factor 11, telomere length, TERT, TERC, telomerase activity, Smad2, replicative cellular senescence

## Abstract

Maintenance of telomere length is essential to delay replicative cellular senescence. It is controversial on whether growth differentiation factor 11 (GDF11) can reverse cellular senescence, and this work aims to establish the causality between GDF11 and the telomere maintenance unequivocally. Using CRISPR/Cas9 technique and a long-term *in vitro* culture model of cellular senescence, we show here that *in vitro* genetic deletion of GDF11 causes shortening of telomere length, downregulation of telomeric reverse transcriptase (TERT) and telomeric RNA component (TERC), the key enzyme and the RNA component for extension of the telomere, and reduction of telomerase activity. In contrast, both recombinant and overexpressed GDF11 restore the transcription of TERT in GDF11^KO^ cells to the wild-type level. Furthermore, loss of GDF11-induced telomere shortening is likely caused by enhancing the nuclear entry of SMAD2 which inhibits the transcription of TERT and TERC. Our results provide the first proof-of-cause-and-effect evidence that endogenous GDF11 plays a causal role for proliferative cells to maintain telomere length, paving the way for potential rejuvenation of the proliferative cells, tissues, and organs.

## Introduction

Growth differentiation factor 11 (GDF11, also known as bone morphogenetic protein 11, BMP11) is a member of transforming growth factor-beta (TGF-β) family. Accumulated evidence has shown that GDF11 is widely expressed in both the proliferative cells and tissues including the activated hepatic stellate cells of the liver (Zhang et al., 2018; Dai et al., 2020; Rochette et al., 2020), keratinocytes and dermal fibroblasts of the skin (Tito et al., 2019; Rochette et al., 2020), pancreas, skeletal muscle, kidney, retina, among others (Simoni-Nieves et al., 2019), and postmitotic cells such as neurons of the central nervous system (Hayashi et al., 2018). The expression of GDF11 in proliferative cells suggests its possible important functions in the proliferative cells (Rochette et al., 2015, 2018). As a potential rejuvenating factor, GDF11 has sparkled unusual interest on its possible role in rejuvenating various organs. Previous studies concerning proliferative organs such as heart, muscle, and the vasculature in the nervous system have created controversial results on whether GDF11 can reverse cellular senescence or not, partly due to its underlying mechanisms have not been clearly established (Loffredo et al., 2013; Katsimpardi et al., 2014; Sinha et al., 2014; Ma et al., 2021). For example, it was found that GDF11 in the blood promotes nerve regeneration using the technique of heterochronic parabiosis (Katsimpardi et al., 2014), improves skeletal muscle function, and reverses ventricular hypertrophy in the aged mice (Sinha et al., 2014), highlighting GDF11 as a promising rejuvenating factor. However, the opposite results were reported later showing that GDF11 inhibits the regeneration of skeletal muscle (Egerman et al., 2015) or GDF11 does not rescue aging-related pathological hypertrophy (Smith et al., 2015). Therefore, the function of GDF11 in the process of aging is still in heated debate. To date, no causality between GDF11 and its effect on replicative cellular senescence has been established unequivocally.

Telomere is located at the end of a chromosome, and it is a complex composed of (TTAGGG)_*n*_ DNA repeat sequence and binding proteins (Chakravarti et al., 2021). Telomere shortens with every division of the cell and the shortening of telomere length will consequently cause replicative cellular senescence, and eventually, lead to organismal aging (Shay and Wright, 2019). It is now generally acknowledged that telomere maintenance is an integrative indicator of the overall health and life span, whereas telomere attrition is an interactive contributor to the progress of aging and age-related diseases in organismal individuals (Martinez and Blasco, 2017; Roake and Artandi, 2020). For proliferative cells, telomere attrition has been established as one of the main contributors to their cellular senescence (Blackburn et al., 2015; Chakravarti et al., 2021). So far, it is still unknown whether endogenous GDF11 plays a causal role in telomere maintenance or attrition. Answering this question is fundamentally important to lay a solid foundation for clarifying the function of GDF11 on replicative cellular senescence.

In this study, we aimed to explore the effect of the endogenous GDF11 on telomere length by using a CRISPR/Cas9 strategy to specifically delete GDF11 gene and a long-term culture cellular senescence model established in this work. Here, we show that *in vitro* deletion of GDF11 in cells caused the shortening of telomere length, downregulation of telomeric reverse transcriptase (TERT), telomeric RNA component (TERC), and reduction of telomerase activity, whereas both the recombinant and overexpressed GDF11 restored the transcription of TERT in GDF11^KO^ cells to the wild-type level. The underlying molecular mechanisms were further explored. Collectively, this work provides the first proof-of-cause-and-effect evidence that loss of GDF11 in proliferative cells accelerates the shortening of telomere length.

## Materials and Methods

### Cell Culture

Neuro-2a cells were cultured in a complete medium at 37°C with 5% CO_2_. The complete medium contains high-glucose DMEM (Gibco, Carlsbad, CA, United States) supplemented with 10% fetal bovine serum (FBS, BI) and 50 IU/ml penicillin/streptomycin (P/S, Gibco, Carlsbad, CA, United States).

### Construction of GDF11 Knockout (GDF11^KO^) Neuro 2a Cell Line

In this work, GDF11 specific gRNA sequences were designed through the designer website^1^ as showed below: 5′-GCCGAAGGTACACCCACAGT-3′, 5′-GAACCGGGTAAGG TAGCTTG-3′. The pX459 plasmids containing different GDF11 sgRNA were cotransfected into Neuro 2a cells by Lipofectamine 2000 (Thermo Fisher Scientific, Waltham, MA, United States) with appropriate cell confluence (>50%). After transfection for 48 h, 3 μg/ml puromycin treatment lasted for 4 days was used to screen antibiotic-resistant Neuro 2a cells. The antibiotic-resistant Neuro 2a cells were resuspended in the complete medium at a cell density of 10 cells/ml culture medium by cell counting. The cells were then further diluted into 96 well plates with 100 μl/well to yield a single cell per well. Monoclonal cell was verified visually under a microscope. After being cultured for approximately 14 days, genomic DNA from GDF11^KO^ and wild type (WT) Neuro 2a cells was extracted by TIANamp genomic DNA kit (TIANGEN, Beijing, China). Primers were designed according to GDF11-specific gRNA sites with primer F (5′-CGCAGAATGGGGAGTCAGAA-3′) and primer R (5′-ATCCTCCACCACCACTTCAG-3′). Polymerase chain reaction (PCR) was performed. Product of PCR should be about 514 bp in GDF11^KO^ Neuro 2a cell and 992 bp in WT Neuro 2a cell.

After verification of genetic deletion of GDF11, single clones of GDF11^KO^ or WT Neuro 2a cell were selected (day 1), cultured, and expended through continuous culture. Cells were passaged once every 3 days and collected at different time points of culture in culture. Cells cultured no more than 10 days were defined as “young” (Y-GDF11^KO^ or Y-WT), whereas those cultured over 65 days were regarded as “old” (O-GDF11^KO^ or O-WT). Three independent clones of both GDF11^KO^ and WT Neuro 2a cells were used in this study.

### Telomere Length Analysis

Telomere length was measured by quantitative telomeric fluorescence *in situ* hybridization (Telo-FISH) with a Cy3-labeled PNA probe (TelC-Cy3 F1002, PNA Bio), as previously described (Ourliac-Garnier and Londono-Vallejo, 2011) with slight modifications. Briefly, Neuro 2a cells were cultured in the presence of colcemid (0.2 mg/ml) for 4 h and were subjected to hypotonic swelling. After fixation in ethanol/acetic acid (3:1, v/v), the cell suspension was dropped onto clean slides and dried overnight. After pepsin treatment, the slides were fixed in 3.7% formaldehyde and then hybridized with Cy3-labeled PNA probe subsequent in 70% formamide for 15 min. The DAPI was used to counterstain chromosomal DNA. Images were obtained by fluorescence microscope (Zeiss Axio Imager M2) and analyzed by MetaSystems to quantify the fluorescence intensity of telomeres. The value of relative telomere fluorescence calculated below 0.01 was categorized as a short telomere.

### RNA-Seq and Data Analysis

Single clone-derived GDF11^KO^ and WT Neuro 2a cells (*n* = 3 per group) cultured for 65 days were prepared for RNA extraction using an RNA isolation kit (Vazyme, Nanjing, China). NEBNext ultra directional RNA library prep kit (NEB, MA, United States) was used to prepare the RNA-seq library. Subsequently, the RNA-seq library was sequenced through NovaSeq 6000 (Illumina, San Diego, CA, United States). Fastq files were passed through FASTQC to control the quality of the data and mapping to the mouse genome (mm 10) was performed by using TopHat (v2.1.1). Differentially expressed genes (DEGs) from GDF11^KO^ and WT Neuro 2a cells were analyzed using the cuffdiff (v2.2.1). Genes with *p* < 0.05 and absolute log_2_ (fold change) ≥ 1 were included in the DEGs. RNA-seq data were deposited in the GEO dataset under ID code GSE167538.

### RNA Extraction and Real-Time PCR

Total RNA of GDF11^KO^ and WT Neuro 2a cells was extracted with RNA isolation kit (Vazyme) and reverse transcribed into cDNA by HiScript SuperMix kit (Vazyme). Quantitative real-time PCR (RT-qPCR) was performed in triplicates in a CFX96 touch thermal cycler (Bio-Rad, Hercules, CA, United States) with SYBR master mix (TsingKe). The designed primers were: GDF11-F 5′-CAAACTGCGGCTCAAGGAG-3′; GDF11-R 5′-TGGGGCTGAAGTGGAAATGA-3′; TERC-F 5′-TCATTAGC TGTGGGTTCTGGT-3′; TERC-R 5′-TGGAGCTCCTGCGCT GACGTT-3′; TERT-F 5′-AGCTGTTTGCTGAGGTGCAG-3′; TERT-R 5′-TACCAGGCTCCACAGGGAA-3′; β-actin-F 5′-CG CAGCCACTGTCGAGT-3′; β-actin-R 5′-CCCACGATGGA GGGGAATAC-3′.

### Telomerase Activity Assay

Relative quantitative telomerase repeat amplification protocol (RQ-TRAP) was performed to determine the relative telomerase activity by calculating the amplified signal. GDF11^KO^ and WT Neuro 2a cells were lysed in telomerase lysis buffer (10 mM Tris–HCl (pH 7.5), 1 mM MgCl_2_, 1 mM EGTA, 0.1 mM PMSF, 5 mM β-mercaptoethanol, 0.5% CHAPS, and 10% glycerol) with RNase free solutions to extract telomerase. Then, the samples were harvested into RNase-free EP tubes and maintained for 30 min on ice. Subsequently, samples were centrifuged at 4°C, 12,000 *g* for 5 min and the supernatants were collected. Protein concentration was determined using BCA protein assay kit (Coolaber, Beijing, China). The reaction system of RQ-TRAP contained 250 ng total protein, 0.8 μl of primer TS (10 μM; 5′-AATCCGTCGAGCAGAGTT-3′), 0.4 μl of primer ACX (10 μM; 5′-GCGCGGCTTACCCTTACCCTTACCCTAACC-3′) and SYBR qPCR master mix (TsingKe) and adjusted to 10 μl of total volume using RNase free H_2_O. The running procedure involved incubation for 30 min at 30°C, followed by incubation for 10 min at 95°C and 40 cycles of PCR at 95°C for 30 s, 60°C for 30 s, and 72°C for 15 s in a CFX96 touch thermal cycler (Bio-Rad, Hercules, CA, United States).

### Recombinant GDF11 Rescue Experiment

WT and GDF11^KO^ Neuro 2a cells were cultured for 65 days and then planted into the 24 well plate. Recombinant GDF11 protein (rGDF11, Peprotech, Cat#120-11) of 100 ng/ml was added into the DMEM medium (Gibco, Carlsbad, CA, United States) without fetal bovine serum of WT and GDF11^KO^ Neuro 2a cells at their appropriate cell confluence (>50%), and the treatment lasted for 24 h. Then, the cells were collected for detecting the transcription of TERT and TERC, and telomerase activity.

### Overexpression of GDF11 in GDF11^KO^ Neuro 2a Cells

To overexpress GDF11 in Neuro 2a cells, pCMV-GDF11-P2A-GFP plasmid or vehicle plasmid (control) was transfected using transfectamine 2000 (Thermo Fisher Scientific Waltham, MA, United States) into GDF11^KO^ Neuro 2a cells which were cultured for 65 days and when they were at appropriate cell confluence (>50%) after plating. After transfection, the Neuro 2a cells were cultured for 48 h and the total RNA was harvested for RT-PCR.

### ChIP-qPCR

Chromatin-immunoprecipitation qPCR (ChIP-qPCR) was performed as described previously (Bhatia et al., 2019) with slight modifications. Briefly, since the transcription of TERC started to reduce at 40 days in culture, we chose cells cultured for 40 days as samples for ChIP-qPCR: the single clone-derived GDF11^KO^ and WT Neuro 2a cells were fixed and cross-linked in 1% formaldehyde for 10 min and quenched by glycine (125 mM) for 5 min at room temperature. Cells were lysed and sonicated to obtain 150–300 bp DNA fragments. IgG isotype (10 μg) or SMAD2 antibody (10 μg Cell Signaling, RRID: AB_10626777) was incubated to immunoprecipitate sonicated chromatin overnight at 4°C. Immunoprecipitated DNA-protein complexes were pulled down with protein A/G beads (Santa Cruz). The beads were sequentially washed using the following washing solutions: low-salt buffer (×1), high-salt buffer (×1), LiCl buffer (×1), and TE buffer (×2). After the final wash, elution buffer (1% SDS, 100 mM NaHCO_3_) was used to resuspend DNA. Deoxyribonucleic acid was reversed cross-linking at 65°C overnight with proteinase K (1 mg/ml). Then, the DNA was purified by using a TIANamp genomic DNA kit (TIANGEN, Beijing, China) and subjected to quantitative PCR (qPCR). The binding motif between SMAD2 and the promotor of TERT or TERC was predicted through the website.^2^ Primers of ChIP-qPCR were designed based on the predicted binding motif. The designed primers of ChIP-qPCR are:

TERC-F 5′-CGGCTTAGAAACTCCCCTAGTC-3′; TERC-R 5′-TGCATATAGCAACTCAGTCACCT-3′; TERT-F 5′-AGAAC TGAGATTGCCACCACC-3′; and TERT-R 5′-GGGACCTAAGG TTTTGCCTGT-3′.

### Statistical Analysis

Statistical analysis was performed using Prism 8 (GraphPad Software, United States). Data were presented as mean ± SEM and subjected to two-tailed unpaired *t*-test one-way ANOVA with Tukey’s test or two-way ANOVA with *post hoc* multiple comparisons of Sidak or Brown-Forsythe ANOVA test with *post hoc* T3 test multiple comparisons of Dunnett when it is appropriate, ^∗^*p* < 0.05, ^∗∗^*p* < 0.01.

## Results

### Loss of GDF11 Shortens Telomere Length in Neuro 2a Cells

It has been recently reported that both the level of GDF11 in the blood and the leukocyte telomere lengths (LTLs) are inversely correlated to the age, and the LTLs are associated with the GDF11 expression level in patients who suffered coronary artery diseases (Opstad et al., 2019), implying that GDF11 is associated with telomere length but no causal evidence has been provided. To explore whether endogenous GDF11 plays a causal role in telomere maintenance or attrition, we deleted GDF11 gene in Neuro 2a cells using CRISPR/Cas9 technique and the genetic deletion of GDF11 was confirmed with RT-PCR (Supplementary Figure 1). To mimic the replicative cellular senescence *in vitro*, we established an *in vitro* replicative cellular senescence model by expanding single clones of GDF11^KO^ or WT Neuro 2a cells in culture, cells were passaged once every 3 days, and collected at different time points of cell culture. Those cells cultured no more than 10 days were defined as “young” (Y-GDF11^KO^ or Y-WT), whereas those cultured over 65 days were regarded as “old” (O-GDF11^KO^ or O-WT). All the results were repeated in two independent subclones. Details of statistical methods, results, and sample sizes of all figures are presented in Supplementary Table 1.

**FIGURE 1:**
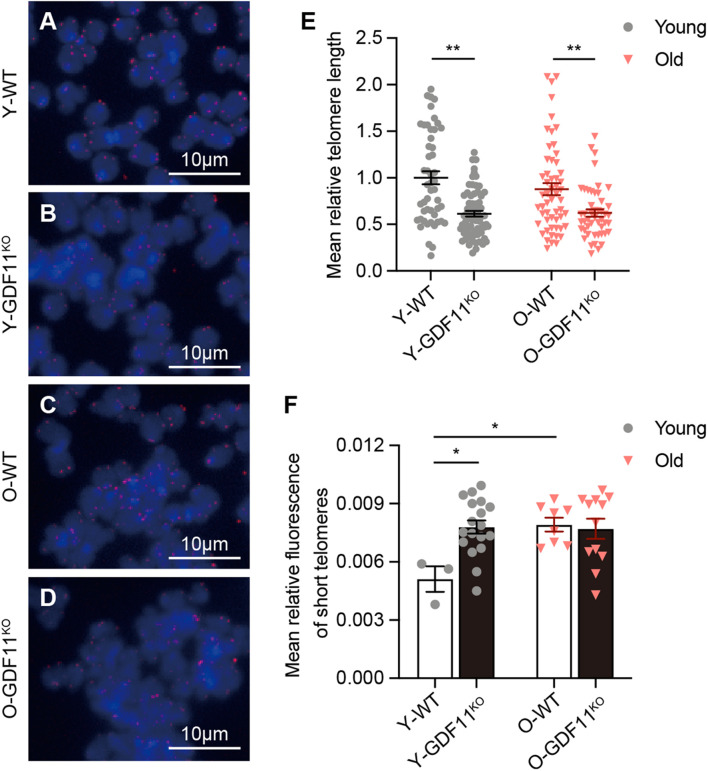
*In vitro* loss of growth differentiation factor 11 (GDF11) shortens telomere length in Neuro 2a cells. Representative images of telomere quantitative fluorescence *in situ* hybridization (Telo-FISH, **A–D**) in single clone-derived GDF11^KO^ or wild type (WT) Neuro 2a cells; young (Y, 10 days in culture), old (O, 65 days in culture). Quantification of the average telomere length **(E)** (two-way ANOVA, interaction: *F*_(1, 217)_ = 1.513, *p* = 0.2201; WT vs. GDF11^KO^: *F*_(1, 217)_ = 37.64, *p* < 0.0001; young vs. old: *F*_(1, 217)_ = 1.183, *p* = 0.2779; Sidak’s test, young: WT vs. GDF11^KO^, *p* < 0.0001; old: WT vs. GDF11^KO^, *p* < 0.0018; Y-WT: *n* = 50, Y-GDF11^KO^: *n* = 68, O-WT: *n* = 66, O-GDF11^KO^: *n* = 48), and the average relative fluorescence of short telomeres **(F)** (two-way ANOVA, interaction: *F*_(1, 37)_ = 6.364, *p* = 0.0161; WT vs. GDF11^KO^: *F*_(1, 37)_ = 4.643, *p* = 0.0378; young vs. old: *F*_(1, 37)_ = 5.642, *p* = 0.0228; Y-WT: *n* = 3, Y-GDF11^KO^: *n* = 18, O-WT: *n* = 8, O-GDF11^KO^: *n* = 12). Data are represented as mean ± SEM, *p < 0.05, **p < 0.01. Scale bars, 10 μm.

Using Telo-FISH (Lai et al., 2018), we further measured the telomere length of GDF11^KO^ and WT cells. Quantification of the frequency of the relative fluorescence intensity of the telomere (Figures 1A–D) showed that in comparison with WT Neuro 2a cells, loss of GDF11 caused a significant shortening of the average telomere length both in the “young” and the “old” groups (Figures 1A–E). Surprisingly, loss of GDF11 indeed caused a significantly increased frequency of short telomere in Y-GDF11^KO^ group in comparison with Y-WT group and reached the “old” level of O-WT (Figure 1F). The average relative fluorescence of the short telomere in the “old” cells was significantly higher than the “young” cells (Figure 1F), suggesting that the long-term cultured cells are suitable for study on telomere length in replicative cellular senescence. Our data provide the first direct evidence that loss of GDF11 shortens telomere length in cells. It is worthwhile to note that loss of GDF11 did not increase short telomere further after cells were cultured over 65 days in O-GDF11^KO^ in comparison with O-WT (Figure 1F), implying that both the “old” GDF11^KO^ and the “old” WT Neuro 2a cells might have reached a senescent state.

### Loss of GDF11 Regulates Telomere Maintenance-Related Genes

**FIGURE 2:**
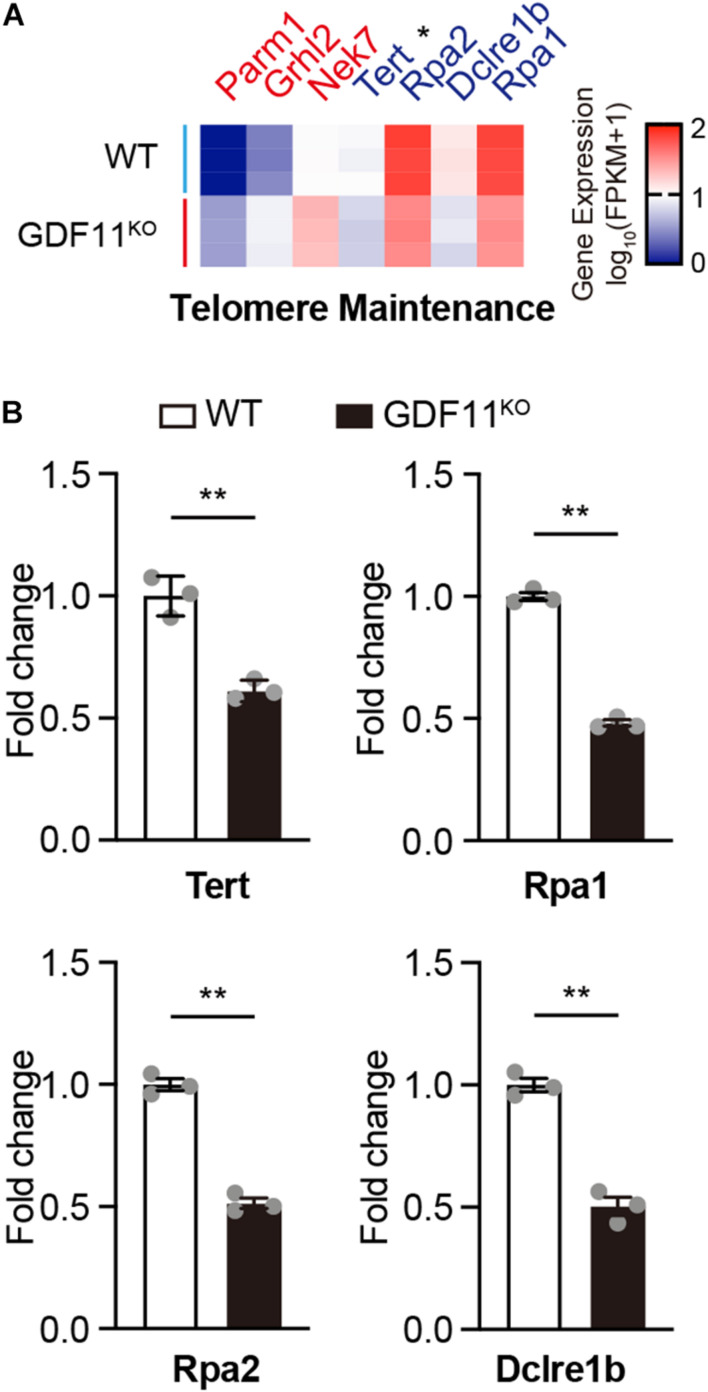
Loss of GDF11 regulates telomere maintenance-related genes. Heatmap of upregulated (3, red) and downregulated (4, blue) **(A)** and bar graphs of 4 downregulated **(B)** telomere maintenance-related genes caused by deletion of GDF11 in single clone derived Neuro2a cells that were cultured for 65 days (two-tailed unpaired *t*-test, Tert: *t* = 7.262, df = 4, *p* = 0.0019; Rpa1: *t* = 24.58, df = 4, *p* < 0.0001; RPA2: *t* = 15.17, df = 4, *p* = 0.0001; DCLRE1b: *t* = 10.63, df = 4, *p* = 0.0004, *n* = 3 per group). Data are represented as mean ± SEM. *p < 0.05, **p < 0.01.

To explore the possible altered genes associated with the telomere maintenance caused by GDF11 knockout, we compared the transcriptome of telomere maintenance relevant genes between GDF11^KO^ and WT Neuro 2a cells by using bulk cells RNA-seq, and these genes were selected from the gene ontology (GO) website.^3^ Our RNA-seq data showed that GDF11 deletion significantly downregulated telomere maintenance genes including TERT, the protein catalytic subunit of telomerase, and replication protein A (Rpa1) and Rpa2 (Figures 2A,B). Our results are consistent with the previous reports that loss of Rpa1 causes telomere shortening (Kobayashi et al., 2010; Aklilu et al., 2020). In addition, our RNA-seq data showed that DNA cross-link repair 1B (Dclre1b) was significantly decreased in the GDF11^KO^ Neuro 2a cells (Figures 2A,B). Dclre1b (also called Apollo) has a role in safeguarding telomeres during the S phase, and loss of Dclre1b causes telomeric deficiencies (Szilard and Durocher, 2006; Schmiester and Demuth, 2017). In addition, loss of GDF11 in Neuro 2a cells regulated genes that are related to cell cycle, and among them, 15 were upregulated whereas six were downregulated (Supplementary Figure 2). Collectively, our data indicated that loss of GDF11 causes shortening of telomere length and this effect is associated with downregulation of TERT and many other telomere maintenance-associated genes.

### Loss of GDF11 Downregulates TERT, TERC, and Telomerase Activity

**FIGURE 3:**
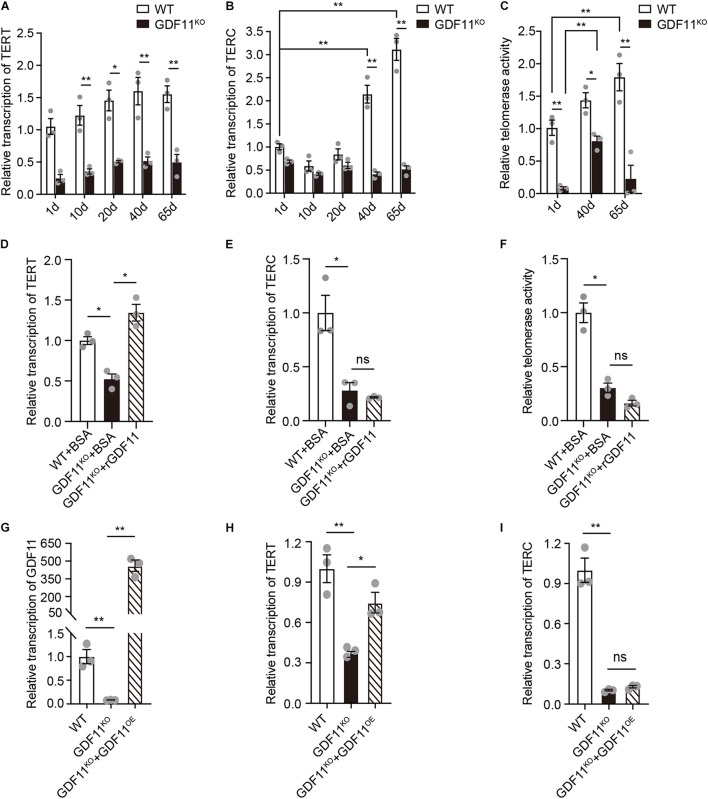
*In vitro* loss of GDF11 downregulates TERT, TERC, and telomerase activity. Quantification of the mRNA level of telomerase reverse transcriptase (TERT, **A**) (two-way ANOVA, interaction: *F*_(4, 20)_ = 0.4232, *p* = 0.7901; WT vs. GDF11^KO^: *F*_(1, 20)_ = 153.1, *p* < 0.0001; time: *F*_(4, 20)_ = 3.983, *p* = 0.0155; Sidak’s test, 1 day: WT vs. GDF11^KO^, *p* = 0.0007; 10 days: WT vs. GDF11^KO^, *p* = 0.0003; 20 days: WT vs. GDF11^KO^, *p* < 0.0001; 40 days: WT vs. GDF11^KO^, *p* < 0.0001; 65 days: WT vs. GDF11^KO^, *p* < 0.0001; *n* = 3 per group) and telomerase RNA component (TERC, **B**) (two-way ANOVA, interaction: *F*_(4, 20)_ = 43.49, *p* < 0.0001; WT vs. GDF11^KO^: *F*_(1, 20)_ = 185.5, *p* < 0.0001; Time: *F*_(4, 12)_ = 39.47, *p* < 0.0001; Sidak’s test, 40 days: WT vs. GDF11^KO^, *p* < 0.0001; 65 days: WT vs. GDF11^KO^, *p* < 0.0001; WT: 1 vs. 40 days, *p* < 0.0001; WT: 1 vs. 65 days, *p* < 0.0001; *n* = 3 per group) as well as the telomerase activity **(C)** (two-way ANOVA, interaction: *F*_(2, 12)_ = 5.662, *p* = 0.0186; WT vs. GDF11^KO^: *F*_(1, 12)_ = 80.51, *p* < 0.0001; time: *F*_(2, 12)_ = 9.197, *p* = 0.0038; Sidak’s test, 1 day: WT vs. GDF11^KO^, *p* = 0.0017; 40 days: WT vs. GDF11^KO^, *p* = 0.0265; 65 days: WT vs. GDF11^KO^, *p* < 0.0001; WT: 1 vs. 65 days, *p* = 0.0067; GDF11^KO^: 1 vs. 40 days, *p* = 0.0104; *n* = 3 per group) in single clone-derived GDF11^KO^ and WT Neuro 2a cells which were cultured for different periods of time. **(D)** Effect of rGDF11 on the transcription of TERT in WT or GDF11^KO^ Neuro 2a cells cultured for 65 days (Brown-Forsythe ANOVA test, Brown-Forsythe ANOVA test: F* (DFn, DFd) = 29.64 (2.000, 4.350), *p* = 0.0029; Welch’s ANOVA test: W (DFn, DFd) = 23.73 (2.000, 3.723), *p* = 0.0076; Dunnett’s T3 test, WT + BSA vs. GDF11^KO^ + BSA, *p* = 0.0109, GDF11^KO^ + BSA vs. GDF11^KO^ + rGDF11, *p* = 0.0159; *n* = 3 per group). **(E)** Effect of rGDF11 on the transcription of telomeric RNA component (TERC) in WT or GDF11^KO^ Neuro 2a cells cultured for 65 days (Brown-Forsythe ANOVA test, Brown-Forsythe ANOVA test: F* (DFn, DFd) = 29.57 (2.000, 3.365), *p* = 0.0073; Welch’s ANOVA test: W (DFn, DFd) = 18.08 (2.000, 2.697), *p* = 0.0274; Dunnett’s T3 test, WT + BSA vs. GDF11^KO^ + BSA, *p* = 0.0326; GDF11^KO^ + BSA vs. GDF11^KO^ + rGDF11, *p* = 0.7786; *n* = 3 per group). **(F)** Effect of rGDF11 on the telomerase activity of WT or GDF11^KO^ Neuro 2a cells cultured for 65 days (Brown-Forsythe ANOVA test, Brown-Forsythe ANOVA test: F* (DFn, DFd) = 55.10 (2.000, 3.288), *p* = 0.0030; Welch’s ANOVA test: W (DFn, DFd) = 34.01 (2.000, 3.400), *p* = 0.0056; Dunnett’s T3 test, WT + BSA vs. GDF11^KO^ + BSA, *p* = 0.0151; GDF11^KO^ + BSA vs. GDF11^KO^ + rGDF11, *p* = 0.1590; *n* = 3 per group). Effects of overexpression of GDF11 **(G)** on transcription of TERT **(H)** and TERC **(I)** in GDF11^KO^ Neuro 2a cells cultured for 65 days. For statistical analysis details, please see Supplementary Table 1. Data are represented as mean ± SEM, *p < 0.05, **p < 0.01.

Telomerase, a ribonucleoprotein complex, is composed of a protein catalytic subunit TERT and an RNA template termed TERC. Telomeric reverse transcriptase gene encodes telomere extending enzyme protein and TERC gene encodes telomerase RNA component, and both TERT and TERC are indispensable for telomere extension (Shippen-Lentz and Blackburn, 1990; Zvereva et al., 2010; Mason et al., 2011). To explore whether deletion of GDF11 affects the transcription of TERT and TERC, quantification of our RT-PCR data showed that the mRNA level of both the protein catalytic subunit TERT and telomerase RNA component TERC significantly decreased in GDF11^KO^ cells (Figures 3A,B). Interestingly, only when Neuro 2a cells were cultured over 40 days, loss of GDF11 decreased the mRNA transcription level of TERC (Figure 3B), suggesting that downregulation of TERC caused by loss of GDF11 is a cumulative process.

Downregulation of TERT and TERC caused by GDF11 deletion could lead to a decrease in telomerase activity. To test this possibility, we used telomere repeat amplification protocol (TRAP) assay, a standard protocol for measuring telomerase activity (Fajkus, 2006). We found that, as expected, the telomerase activity in both “young” and “old” GDF11^KO^ Neuro 2a cells showed a sharp decrease in comparison with that of WT Neuro 2a cells (Figure 3C).

To test whether loss of GDF11 induced downregulation of TERT, TERC, and telomerase activity can be restored by adding recombinant GDF11 (rGDF11), we showed that 24 h rGDF11 treatment to the GDF11^KO^ Neuro 2a cells indeed restored the transcription of TERT to the level equivalent to the WT (Figure 3D). However, rGDF11 did not affect the transcription of TERC (Figure 3E) or the telomerase activity (Figure 3F), suggesting that rGDF11 at least partially restored the phenotypes caused by genetic deletion of GDF11. Similarly, overexpression of GDF11 in GDF11^KO^ cells (Figure 3G) also restored the transcription of TERT to the WT level (Figure 3H) but did not affect the transcription of TERC (Figure 3I). Our results provide direct evidence that loss of GDF11 *in vitro* causes telomere length shortened accompanied with downregulation of TERT and TERC and decrease of telomerase activity.

### Loss of GDF11 Enhances SMAD2 Binding to the Promoter of TERT and TERC

**FIGURE 4:**
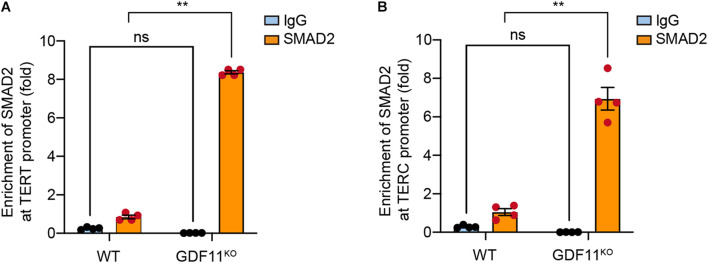
Loss of GDF11 enhances SMAD2 binding to the promoters of TERT and TERC. CHIP-qPCR assessment of the enrichment of SMAD2 at the promoters of TERT **(A)** (two-way ANOVA, interaction: *F*_(1, 12)_ = 3,595, *p* < 0.001; WT vs. GDF11^KO^: *F*_(1, 12)_ = 3,176, *p* < 0.001; IgG vs. SMAD2: *F*_(1, 12)_ = 4,788, *p* < 0.001; Sidak’s test, SMAD2: WT vs. GDF11^KO^, *p* < 0.001; four samples per group) and TERC **(B)** (two-way ANOVA, interaction: *F*_(1, 12)_ = 101, *p* < 0.001; WT vs. GDF11^KO^*: F*_(1, 12)_ = 83.6, *p* < 0.001; IgG vs. SMAD2: *F*_(1, 12)_ = 157, *p* < 0.001; Sidak’s test, SMAD2: WT vs. GDF11^KO^, *p* < 0.001; four samples per group) in the single clone-derived GDF11^KO^ and WT Neuro 2a cells that were cultured for 40 days. Data are represented as mean ± SEM, **p < 0.01.

Since SMAD2 is a key molecular mediator for the GDF11 signaling pathway (Rochette et al., 2015; Duran et al., 2018), we wonder whether SMAD2 directly binds to the promoter of TERT or TERC. We performed ChIP-qPCR and showed that SMAD2 was significantly enriched at the promoters of both TERT (Figure 4A) and TERC (Figure 4B) in GDF11^KO^ Neuro 2a cells, suggesting that SMAD2 can bind to the promoters of both TERT and TERC. Since we found that loss of GDF11 caused downregulation of TERT and TERC and enhanced nuclear entry of SMAD2, the enrichment of SMAD2 at the promoters of TERT and TERC could inhibit their transcription. Collectively, based on our results and previous reports, we propose here that loss of GDF11 enhances SMAD2 binding to the promoters of TERT and TERC and inhibits the transcription of TERT and TERC and eventually causes shortening of telomere and reduction of telomerase activity.

## Discussion

Growth differentiation factor 11 has attracted intensive attention in the recent years as a potential rejuvenation factor (Loffredo et al., 2013; Katsimpardi et al., 2014; Sinha et al., 2014). However, recent studies have reported conflicting results regarding the role of GDF11 on rejuvenation (Ma et al., 2021). This controversy suggests that the functional roles of GDF11 are complicated, and revealing the fundamental role of endogenous GDF11 could provide clear clues for clarification of the GDF11 function. Growth differentiation factor 11 has been shown widely expressed in proliferative cells and tissues (Zhang et al., 2018; Simoni-Nieves et al., 2019; Tito et al., 2019; Dai et al., 2020; Rochette et al., 2020). However, it is still unknown whether GDF11 plays a causal role in replicative cellular senescence.

This study aimed to explore whether endogenous GDF11 plays a causal role in the maintenance or attrition of telomere. To answer this question, using the CRISPR/Cas9 technique and a long-term *in vitro* culture model of cellular senescence, we specifically knocked out GDF11 gene in the Neuro 2a cell line. We used a standard method to measure the telomere length (Lansdorp et al., 1996; Lai et al., 2018), we measured the frequency of relative fluorescence intensity of foci, the shortest telomeres in single cells and the average telomere length at each chromosome end with higher accuracy. Our results consistently showed that loss of GDF11 shortens telomere length in Neuro 2a cells.

To explore the mechanisms underlying the shortening of telomere length caused by loss of GDF11, we performed bulk cells RNA-seq. We found that accompanied with shortening of telomere length, loss of GDF11 in Neuro 2a cells caused downregulation of many telomere maintenances associated genes such as Rpa1 (Kobayashi et al., 2010; Aklilu et al., 2020) and Dclre1b (also called Apollo) (Szilard and Durocher, 2006; Schmiester and Demuth, 2017). These results consistently support that loss of GDF11 shortens telomere length and suggest the possible underlying molecular mechanisms.

Progressive telomere erosion in the TERT or TERC null mice contributes to organ atrophy in highly proliferative tissues including regenerating injured liver, testis, bone marrow, spleen, and blood (Lee et al., 1998; Rudolph et al., 2000) indicating that for the highly proliferative tissues it is very important to maintain telomere length. Based on the previous reports, telomerase is the most common mechanism underlying the telomere maintenance in highly proliferative tissues. A decrease of telomerase activity will cause the shortening of telomere length, therefore, the regulation of the telomerase activity is crucial to maintain telomere length throughout the cellular life span (Chakravarti et al., 2021). Detecting the mRNA levels of TERT and TERC together with the telomerase activity assay (RQ-TRAP) is a set of reliable biomarkers to determine the telomerase activity (Bodnar et al., 1998). However, the molecular mechanisms underlying the maintenance of telomere length remain poorly understood. Our RNA-seq and RT-PCR data showed that loss of GDF11 in Neuro 2a cells caused downregulation of TERT, the telomere extending enzyme, and TERC, the telomerase RNA component, both of which are essential for extension of telomere (Shippen-Lentz and Blackburn, 1990; Zvereva et al., 2010; Mason et al., 2011). Furthermore, we found that the telomerase activity in GDF11^KO^ Neuro 2a cells showed a sharp decrease in comparison with that of WT Neuro 2a cells. In contrast, both recombinant and overexpressed GDF11 restore the transcription of TERT in GDF11^KO^ cells to the WT level but do not affect transcription of TERC. Collectively, our results provide the first direct evidence that loss of GDF11 *in vitro* causes telomere length shortened accompanied with downregulation of TERT and TERC and reduction of telomerase activity.

The biological effect of GDF11 is shaped by the involved signaling pathways, and SMAD2 is one of the key mediators in the well-accepted central signaling pathway activated by GDF11 (Rochette et al., 2015; Duran et al., 2018). Growth differentiation factor 11 acts as one of the TGFβ family ligands that bind to activin type II receptor and activate activin type I receptor. The type I receptor subsequently phosphorylates SMAD2 or SMAD3, then the SMAD complex bind with SMAD4 and then translocate into the nucleus (Gaunt et al., 2013; Bajikar et al., 2017). Based on these, we ask whether SMAD2 causes downregulation of TERT and TERC in GDF11^KO^ Neuro 2a cells. As expected, our data showed that loss of GDF11 enhances SMAD2 binding to the promoters of TERT and TERC, and loss of GDF11 enhances nuclear entry of SMAD2, suggesting that telomere shortening induced by loss of GDF11 is likely caused by SMAD2 inhibiting the transcription of TERT and TERC. Previous reports indicated that the phosphorylated SMAD3 is elevated markedly in the lung of G3 TERC^–/–^ mice (Liu et al., 2018), and SMAD3 binds directly to the promoter of TERT gene and induces TERT gene repression (Li and Liu, 2007; Cassar et al., 2010). By inhibiting phosphorylation of SMAD2/3, which prevents repression of TERT, Pim1 maintains telomere lengths in cardiomyocytes (Ebeid et al., 2021). Our results indicate that GDF11 maintains telomere length in Neuro 2a cells, unlike TGF-β which shortens telomere in cancer cells (Li and Liu, 2007; Cassar et al., 2010). Due to the vital role and complicated mechanisms underlying telomere length maintenance, the different effects among various members of TGF-β family deserve further studies, and it is possible that different members of TGF-β family function in biological contexts dependent manners. For the first time, our data show that the endogenous GDF11 plays a causal role for proliferative cells to maintain telomere length, and this effect is likely mediated by SMAD2, which can bind to the promoters of TERT and TERC. Our work lays a solid foundation that GDF11 plays an essential role in maintaining telomere length in replicative cellular senescence.

Using Neuro 2a cell, a highly proliferative cell line, our long-term culture results showed that loss of GDF11 shortens telomere length. Interestingly, GDF11 knock-out induced a decrease in the average relative fluorescence of short telomere and the relative transcription of TERC took place in a time-dependent manner. These results suggest that the long-term cultured proliferative cell is a suitable *in vitro* model for studying telomere length on replicative cellular senescence.

In summary, we show here that *in vitro* genetic ablation of GDF11 causes shortening of telomere length, downregulation of TERT and TERC, the key enzyme, and the RNA component for extension of telomere, respectively, and reduction of telomerase activity in a long-term cultured Neuro 2a cells. Furthermore, we also found that telomere shortening induced by loss of GDF11 is likely caused by SMAD2 inhibiting the transcription of TERT and TERC. Our results provide the first proof-of-cause-and-effect evidence that endogenous GDF11 plays a causal role for proliferative cells to maintain telomere length in replicative cellular senescence, paving the way for potential rejuvenation of the proliferative cells, tissues, and organs by manipulating GDF11.

## Data Availability Statement

The datasets presented in this study can be found in online repositories. The names of the repository/repositories and accession number(s) can be found below: https://www.ncbi.nlm.nih.gov/geo/, GSE167538.

## Author Contributions

J-WZ defined the topic of this project. J-WZ and D-XW designed the experiments and wrote the manuscript. D-XW, X-DZ, and X-RM performed the experiments. D-XW, X-DZ, X-RM, Z-JD, R-RL, and Y-NC analyzed the data. L-BW provided some resources. All authors have read and approved the final manuscript.

## Conflict of Interest

The authors declare that the research was conducted in the absence of any commercial or financial relationships that could be construed as a potential conflict of interest.

## Publisher’s Note

All claims expressed in this article are solely those of the authors and do not necessarily represent those of their affiliated organizations, or those of the publisher, the editors and the reviewers. Any product that may be evaluated in this article, or claim that may be made by its manufacturer, is not guaranteed or endorsed by the publisher.

## Footnotes

1
https://chopchop.cbu.uib.no/

2
http://jaspar.genereg.net/

3
http://www.informatics.jax.org/

## Supplementary Material

Supplementary Table 1 Details of statistical methods, results, and sample sizes of all figures are presented.
