# Supplementary material for: Loss of Growth Differentiation Factor 11 Shortens Telomere Length by Downregulating Telomerase Activity
Source: Front Physiol. 2021 Sep 13;12:726345. doi: 10.3389/fphys.2021.726345 (PMC8473905; doi:10.3389/fphys.2021.726345)
Supplement: Supplementary Table 1 — Details of statistical methods, results, and sample sizes of all figures are presented. [file Table_1.pdf]

Supplementary Table . Statistical methods, results and sample size related to each figure.

Figure	Statistical method	Factors	Statistical results	*Post hoc* method	*Post hoc* results	sample size
1E	Two-way ANOVA	WT versus $GDF11^{KO}$;<br>Young versus Old	Interaction: $F_{(1,217)}$ = 1.513, P = 0.2201;<br>WT versus $GDF11^{KO}$: $F_{(1,217)}$ = 37.64, P< 0.0001;<br>Young versus Old: $F_{(1,217)}$ = 1.183, P = 0.2779	Sidak's test	Young: WT versus $GDF11^{KO}$, P < 0.0001;<br>Old: WT versus $GDF11^{KO}$, P < 0.0018;	Y-WT: n = 50;<br>Y-$GDF11^{KO}$: n = 68;<br>O-WT: n = 66;<br>O-$GDF11^{KO}$: n = 48;
1F	Two-way ANOVA	WT versus $GDF11^{KO}$;<br>Young versus Old	Interaction: $F_{(1,37)}$ = 6.364, P = 0.0161;<br>WT versus $GDF11^{KO}$: $F_{(1,37)}$ = 4.643, P = 0.0378;<br>Young versus Old: $F_{(1,37)}$ = 5.642, P = 0.0228	Sidak's test	Young: WT versus $GDF11^{KO}$, P = 0.0125;<br>WT: Young versus Old, P = 0.0163;	Y-WT: n = 3;<br>Y-$GDF11^{KO}$: n = 18;<br>O-WT: n = 8;<br>O-$GDF11^{KO}$: n = 12;
2B	Two-tailed unpaired *t* test	WT versus $GDF11^{KO}$	Tert: t = 7.262, df = 4, P = 0.0019;<br>Rpa1: t = 24.58, df = 4, P < 0.0001;<br>Rpa2: t = 15.17, df = 4, P = 0.0001;<br>Dclre1b: t = 10.63, df = 4, P = 0.0004;			3 samples per group
3A	Two-way ANOVA	WT versus $GDF11^{KO}$;<br>Time	Interaction: $F_{(4,20)}$ = 0.4232, P = 0.7901;<br>WT versus $GDF11^{KO}$: $F_{(1,20)}$ = 153.1, P < 0.0001;<br>Time: $F_{(4,20)}$ = 3.983, P = 0.0155	Sidak's test	1 d: WT versus $GDF11^{KO}$, P = 0.0007;<br>10 d: WT versus $GDF11^{KO}$, P = 0.0003;<br>20 d: WT versus $GDF11^{KO}$, P < 0.0001;<br>40 d: WT versus $GDF11^{KO}$, P < 0.0001;<br>65 d: WT versus $GDF11^{KO}$, P < 0.0001	3 samples per group
3B	Two-way ANOVA	WT versus $GDF11^{KO}$;<br>Time	Interaction: $F_{(4,20)}$ = 43.49, P < 0.0001;<br>WT versus $GDF11^{KO}$: $F_{(1,20)}$ = 185.5, P < 0.0001;<br>Time: $F_{(4,12)}$ = 39.47, P < 0.0001	Sidak's test	40 d: WT versus $GDF11^{KO}$, P < 0.0001;<br>65 d: WT versus $GDF11^{KO}$, P < 0.0001;<br>WT : 1 d versus 40 d, P < 0.0001;<br>WT : 1 d versus 65 d, P < 0.0001;	3 samples per group
3C	Two-way ANOVA	WT versus $GDF11^{KO}$;<br>Time	Interaction: $F_{(2,12)}$ = 5.662, P = 0.0186;<br>WT versus $GDF11^{KO}$: $F_{(1,12)}$ = 80.51, P < 0.0001;<br>Time: $F_{(2,12)}$ = 9.197, P = 0.0038	Sidak's test	1 d: WT versus $GDF11^{KO}$, P = 0.0017;<br>40 d: WT versus $GDF11^{KO}$, P = 0.0265;<br>65 d: WT versus $GDF11^{KO}$, P < 0.0001;<br>WT: 1 d versus 65 d, P = 0.0067;<br>$GDF11^{KO}$: 1 d versus 40 d, P = 0.0104;	3 samples per group
3D	Brown-Forsythe ANOVA test	Condition	Brown-Forsythe ANOVA test: F* (DFn, DFd) = 29.64 (2.000, 4.350), P = 0.0029; Welch's ANOVA test: W (DFn, DFd)= 23.73 (2.000, 3.723) , P = 0.0076	Dunnett's T3 test	WT+BSA versus $GDF11^{KO}$+BSA, P = 0.0109;<br>$GDF11^{KO}$+BSA versus $GDF11^{KO}$+rGDF11, P = 0.0159	3 samples per group
3E	Brown-Forsythe ANOVA test	Condition	Brown-Forsythe ANOVA test: F* (DFn, DFd) = 29.57 (2.000, 3.365), P = 0.0073; Welch's ANOVA test: W (DFn, DFd)= 18.08 (2.000, 2.697), P = 0.0274	Dunnett's T3 test	WT+BSA versus $GDF11^{KO}$+BSA, P = 0.0326;<br>$GDF11^{KO}$+BSA versus $GDF11^{KO}$+rGDF11, P = 0.7786	3 samples per group
3F	Brown-Forsythe ANOVA test	Condition	Brown-Forsythe ANOVA test: F* (DFn, DFd) = 55.10 (2.000, 3.288), P = 0.0030; Welch's ANOVA test: W (DFn, DFd)= 34.01 (2.000, 3.400) , P = 0.0056	Dunnett's T3 test	WT+BSA versus $GDF11^{KO}$+BSA, P = 0.0151;<br>$GDF11^{KO}$+BSA versus $GDF11^{KO}$+rGDF11, P = 0.1590	3 samples per group
3G	Two-tailed unpaired *t* test	Condition	WT versus $GDF11^{KO}$: t = 6.070, df = 4, P = 0.0037; $GDF11^{KO}$ versus $GDF11^{OE}$: t = 9.416, df = 4, P = 0.0007;			3 samples per group
3H	One-way ANOVA	Condition	F (DFn, DFd) : F (2, 6) = 17.70, P = 0.0030	Tukey's test	WT versus $GDF11^{KO}$, P = 0.0025 ;<br>$GDF11^{KO}$ versus $GDF11^{OE}$, P = 0.0285	3 samples per group
3I	One-way ANOVA	Condition	F (DFn, DFd) : F (2, 6) = 92.29, P < 0.0001	Tukey's test	WT versus $GDF11^{KO}$, P < 0.0001 ;<br>$GDF11^{KO}$ versus $GDF11^{OE}$, P = 0.9366	3 samples per group
4A	Two-way ANOVA	WT versus $GDF11^{KO}$;<br>IgG versus SMAD2	Interaction: $F_{(1,12)}$ = 3595, P < 0.001;<br>WT versus $GDF11^{KO}$: $F_{(1,12)}$ = 3176, P < 0.001;<br>IgG versus SMAD2: $F_{(1,12)}$ = 4788, P < 0.001	Sidak's test	SMAD2: WT versus $GDF11^{KO}$, P<0.001	4 samples per group
4B	Two-way ANOVA	WT versus $GDF11^{KO}$;<br>IgG versus SMAD2	Interaction: $F_{(1,12)}$ = 101, P < 0.001;<br>WT versus $GDF11^{KO}$: $F_{(1,12)}$ = 83.6, P < 0.001;<br>IgG versus SMAD2: $F_{(1,12)}$ = 157, P < 0.001	Sidak's test	SMAD2: WT versus $GDF11^{KO}$, P<0.001	4 samples per group
