# Supplementary figures and images for: Loss of Growth Differentiation Factor 11 Shortens Telomere Length by Downregulating Telomerase Activity
Source: Front Physiol. 2021 Sep 13;12:726345. doi: 10.3389/fphys.2021.726345 (PMC8473905; doi:10.3389/fphys.2021.726345)

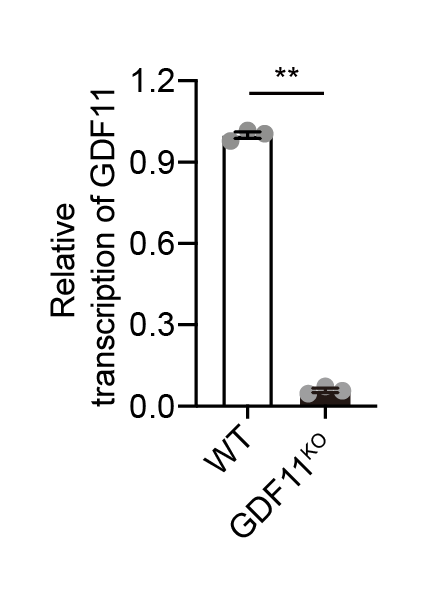

Supplement: Supplementary file 2 [file Image_1.TIF]

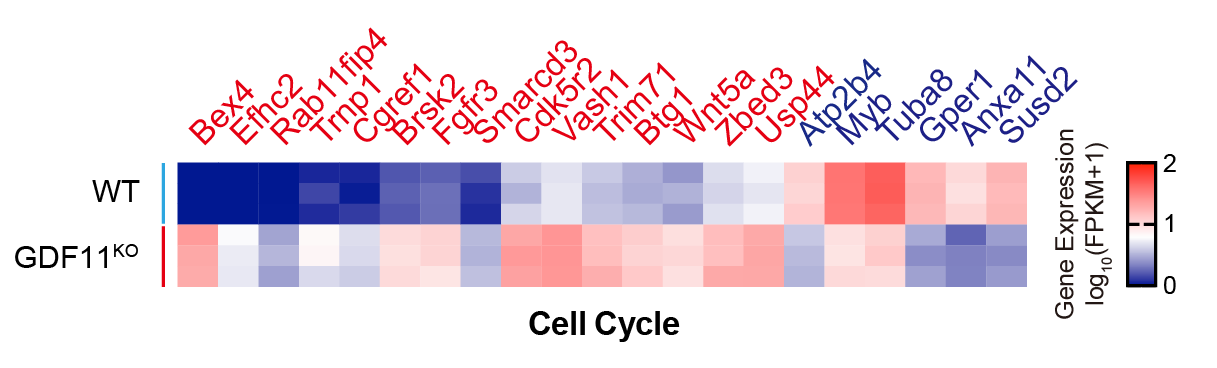

Supplement: Supplementary file 3 [file Image_2.TIF]
